# Electrochemical Performance of Supercapacitor with Stacked Copper Foils Coated with Graphene Nanoplatelets

**DOI:** 10.1038/s41598-018-21572-x

**Published:** 2018-02-15

**Authors:** S. L. Chiam, H. N. Lim, S. M. Hafiz, A. Pandikumar, N. M. Huang

**Affiliations:** 1 Department of Chemistry, Faculty of Science, Universiti Putra Malaysia, 43400 UPM Serdang, Selangor Malaysia; 2 Materials Synthesis and Characterization Laboratory, Institute of Advanced Technology, Universiti Putra Malaysia, 43400 UPM Serdang, Selangor Malaysia; 3 Electrochemical Materials Science and Functional Materials Division, CSIR-Central Electrochemical Research Institute, Karaikudi, 630003 India; 4 New Energy Science & Engineering Programme, University of Xiamen Malaysia, Jalan SunSuria, Bandar SunSuria, 43900 Sepang, Selangor Darul Ehsan Malaysia

## Abstract

The energy density of conventional supercapacitors is in the range of 6–10 Wh kg^−1^, which has restricted them from many applications that require devices with long durations. Herein, we report a method for enhancing the energy density of a device through the parallel stacking of five copper foils coated on each side with graphene nanoplatelets. Microporous papers immersed in 2 M aqueous sodium sulphate were used as separators. With a low contact resistance of 0.05 Ω, the supercapacitor yielded an optimum specific energy density and a specific power density of 24.64 Wh kg^−1^ and 402 W kg^−1^ at 0.8 V, respectively. The working potential was increased to 2.4 V when three of the supercapacitors were connected in series, forming a tandem device. Its potential for real applications was manifested by the ability to light up a light-emitting diode for 40 s after charging for 60 s.

## Introduction

In the past two decades, there has been rapid growth in the study of supercapacitors^1–8^. This has primarily been the result of two attractive characteristics, their fast charge/discharge rates (a few seconds to milliseconds) and long life cycles (usually more than 10^4^ cycles)^9,10^. Hence, these devices can complement or replace batteries when high power delivery or long cycling stability is required, or there is a need for intermittent energy with variable power demands, such as in the case of electrical vehicles during accelerating and braking. Yet, despite their promising features, the prevalent use of supercapacitors is still restricted by a primary challenge that needs to be overcome before they can be developed as advanced energy storage devices. This challenge is their energy density. In general, the energy densities of supercapacitors (6–10 Wh kg^−1^) are still far from satisfactory compared to those of Li-ion batteries (60–100 Wh kg^−1^)^11^.

To achieve a higher charge storage capability, electrode materials with highly accessible surface areas and excellent conductivities are mandatory. As a unique material, graphene nanoplatelets (GNPs), which consist of short stacks of graphene sheets, are an excellent matching candidate because of their ability to display superlative two-dimensional characteristics, which include good electronic properties, a high accesible surface area, and superior mechanical properties^12–14^. Furthermore, the inherently rapid energy storage mechanism of GNPs, which only involves the simple movement of ions back and forth on the electrode surface, contributes to a system with high power density. The unique structural characteristics and remarkable properties of GNPs contribute to a stable charge storage system with fast ionic transport.

Nonetheless, an individual supercapacitor cell is incapable of single-handedly providing high energy storage comparable to that provided by batteries. Thus, to achieve a high energy density required by many power devices, an effective and simple approach is to stack cells in parallel. This approach is commonly used for batteries^15,16^, but has only been applied in a few cases of symmetrical supercapacitors with a solid electrolyte^11,17^ and acetonitrile organic electrolyte^18^. However, solid-state supercapacitors suffer from poor rate capability as a result of the limited ion-diffusion rate of solid-state electrolytes and large contact resistance between the electrodes and electrolyte interface^19^. At the same time, an acetonitrile organic electrolyte is controversial because of its harmful effects on the environment and humans^20^. To date, no supercapacitors with stacked cells using an aqueous electrolyte have been investigated.

In this work, a supercapacitor was assembled using five copper foils coated on each side with GNPs and separated by microporous papers immersed in 2 M aqueous sodium sulphate. This paper highlights the electrochemical performance of a supercapacitor with multiple cells for plausible real applications by comparing it to a commercially available supercapacitor.

## Results and Discussion

**Figure 1:**
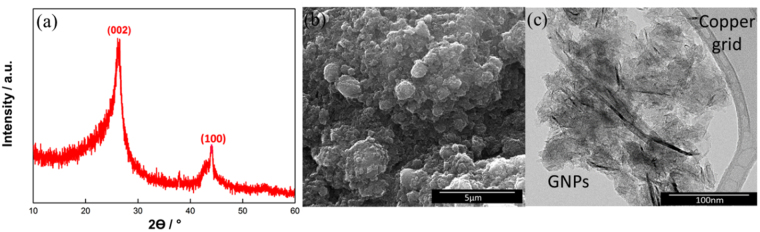
(**a**) XRD (**b**) FESEM, and (**c**) TEM images of GNPs.

The XRD pattern of the GNPs in Fig. 1a shows a prominent peak at 25.6°, indicative of an interlayer spacing of ~3.4 Å between the (002) graphitic crystal planes in GNPs^11^. The small peak at 42.2° was assigned to the (100) plane of the GNPs. Figure 1b shows a high magnification FESEM image, where the GNPs interact with each other and form aggregates. Compared to porous materials with a high surface area, the surface area of GNPs does not depend on the distribution of the pore size. Hence, even in the presence of aggregates, GNPs, which are compliant with high aspect ratio platelets, can physically “move” to improve the accessibility of their surfaces to electrolytes^21,22^. Figure 1c displays corrugated sheets of GNPs, which is the intrinsic nature of GNPs. These make it possible for 2D nanostructures to be thermodynamically stable during a bending or twisting process^23,24^.

**Figure 2:**
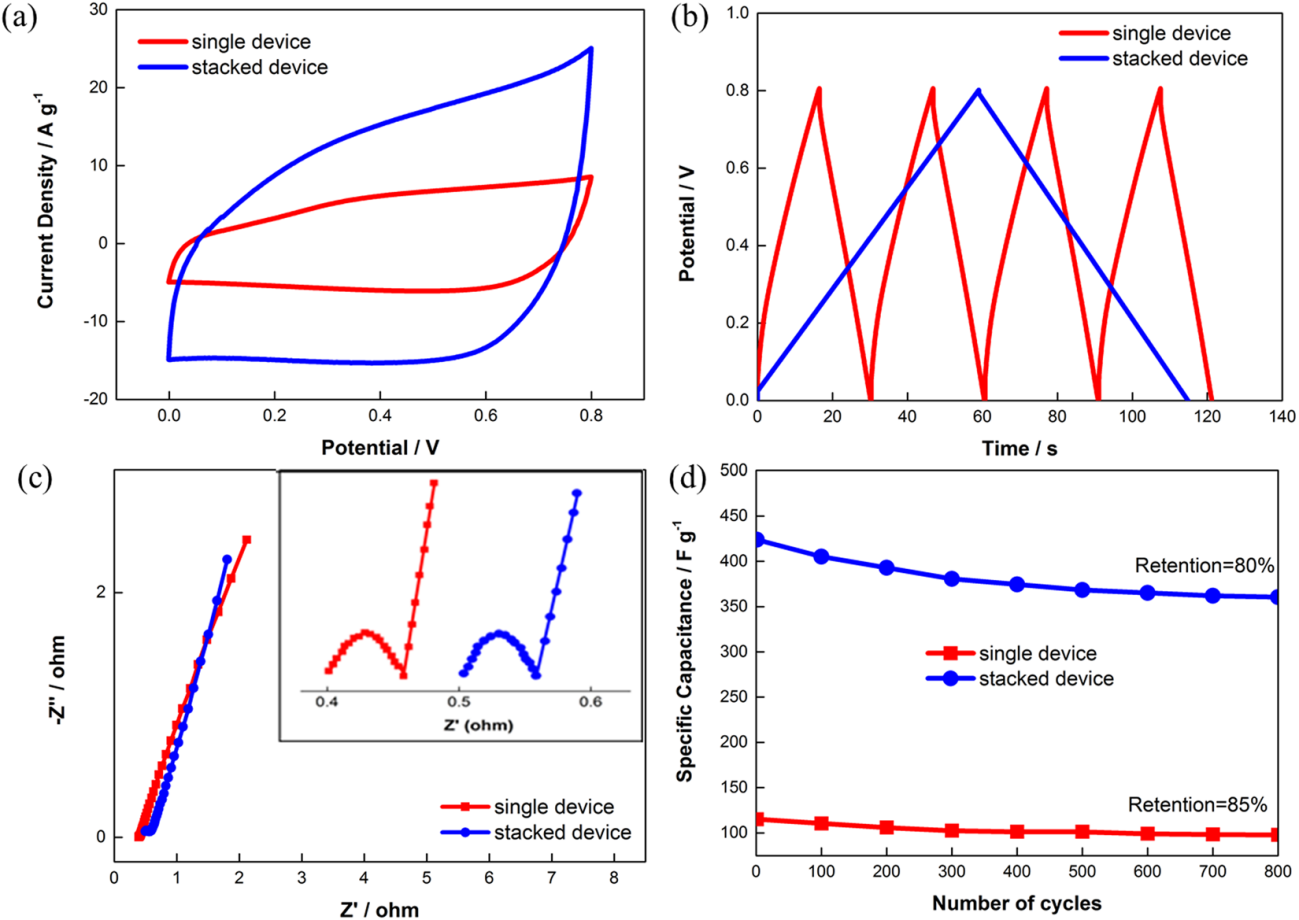
Electrochemical performance of stacked device against single device: (**a**) CV profiles at 100 mV s^−1^, (**b**) GCD profiles at 3 A g^−1^, (**c**) Nyquist plot with inset showing magnified version at low-frequency region, and (**d**) performance durability test at 3 A g^−1^.

A Brunauer–Emmett–Teller (BET) analysis **(**Fig. 2**)** has been conducted to determine the surface area and pore size distribution of GNP. In contrast to a single layer of graphene, GNP is a particle consisting of multiple layers of graphene. The surface area measurement of the GNPs via nitrogen gas absorption yielded a BET value of 591 m^2^/g. This value is 213 times higher compared to graphitic powder (2.78 m^2^/g) 6, indicating that graphite was significantly exfoliated into few graphitic layers (2630/591 ≈ 4) by thermal exfoliation. The adsorption isotherm of GNPs is type IV, which is typically associated with mesopores (50 nm > mesopores > 2 nm). From the BET measurement, the average pore width of GNPs is 62.5 Å, where this pore size allows the easy movement of the Na^+^ (1.02 Å) and SO_4_^2−^ (2.58 Å) electrolyte back and forth on the electrode surface, hence contributing to the capacitance of the supercapacitor.

**Figure 3:**
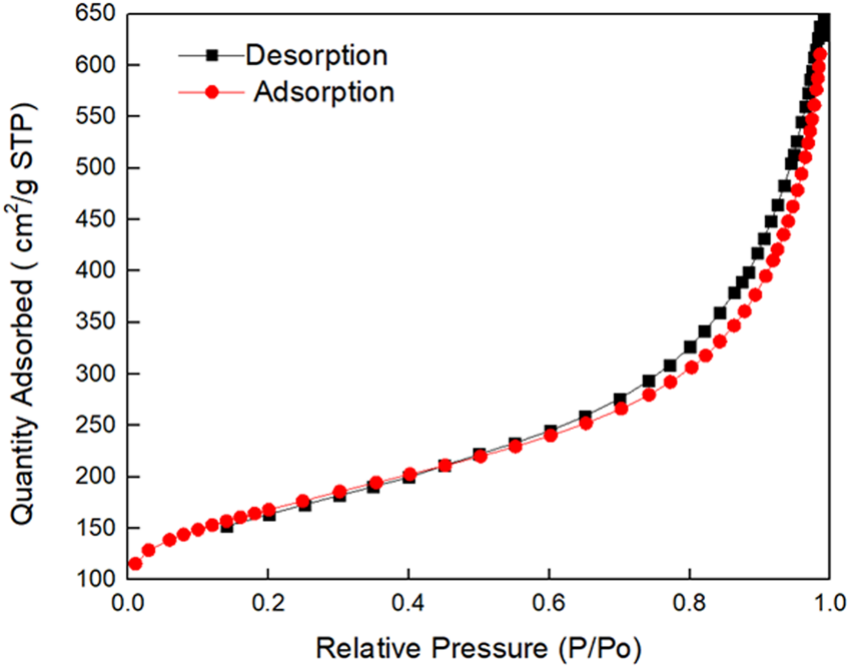
Nitrogen adsorption and desorption isotherms for GNPs.

Figure 3a presents the CV curves of the stacked device at a high scan rate of 100 mV s^−1^ in a potential window of 0−0.80 V. A typical pseudorectangular shape is observed, demonstrating the well maintained electrochemical double layer behavior in the stacked configuration. Moreover, the area under the curve of the stacked device is remarkably higher than that of a supercapacitor assembled with only two copper foils coated with GNPs, which is called a single device. This demonstrated that the stacked device contained more electroactive species, and thus provided an extra charge for the storage mechanism. Consistently, as shown in Fig. 3b, the energy density calculated from the GCD curves of the stacked device increased in magnitude fourfold to 24.64 Wh kg^−1^ compared to the 6.28 Wh kg^−1^ achieved by a single device. This value complements the fourfold discharge time of the stacked device, indicating that a fourfold amount of capacitance was stored. To demonstrate the remarkable durability of a stacked device over a wide range of scan rates and current densities, additional CV and GCD curves at various scan rates and current densities are shown in Figure S1. More importantly, even though there was an increase in the contact layer between the electrode materials and separators, the stacked supercapacitor presented linear and symmetrical GCD profiles with a negligible IR drop. This implied the low resistivity of the GNP electrodes to mass transfer and also the excellent charge propagation of ions at the interfaces between the electrolyte and GNPs, which were contributed by the unique morphology of the agglomerated GNPs^25,26^.

To further understand the resistivity encountered by the stacked device during ionic transport at the electrode and electrolyte interface, electrochemical impedance spectroscopy (EIS) was conducted. As shown in the Nyquist plot depicted in Fig. 3c, a typical curve is observed, whereby a semicircle formed at the high-frequency region, followed by a steep straight line in the low-frequency region. This has been demonstrated to be an ideal circuit with a single time constant and fast ion diffusion rate^27^. At the same time, the equivalent series resistance (ESR) value, which considered the contact resistance between the electrode and electrolyte, could be obtained at the first intercepting point at the x-axis. The ESR value of the stacked device remained low at approximately 0.50 Ω (vs. 0.4 Ω for a single device), suggesting that with an appropriate and tightened configuration design, the resistivity of a stacked device would be insignificant. The low ESR value was in agreement with the ideal charge-discharge curve shown in Fig. 3b. On the other hand, the diameter of the semicircle in the inset of Fig. 3c represents the interfacial charge-transfer resistance (Rct) at the electrode and electrolyte interface. The stacked device recorded an Rct value of 0.03 Ω, which was similar to that of the single device, indicating a low charge transfer resistance despite the increased number of cells. The low Rct value was mainly related to the distinctive characteristics of GNPs, where the ions in the electrolyte could easily migrate throughout the interstices of the agglomerated GNP network and access the large internal surface area of the GNPs, which facilitated the rapid charge transportation and stabilization of the overall system^21^. Therefore, the ideal capacitive behavior of the GNPs was inherent in the stacked device, simplifying the scaling up process for real applications. Moreover, the intimate integration of electrode materials with the copper foil substrate and the high mobility of the aqueous electrolyte in direct contact with the electrode materials were crucial in determining the electrochemical performance of the stacked device^28,29^. Lastly, the cyclic retention and stability were determined under a current density of 3 A g^−1^, as depicted in Fig. 3d. The stacked device retained 80% of its initial capacitance. Although there was a 5% capacitance drop in the stacked device compared to that of the single device, it was compensated by the increase in the specific capacitance value, which broadened its practical application.

**Figure 4:**
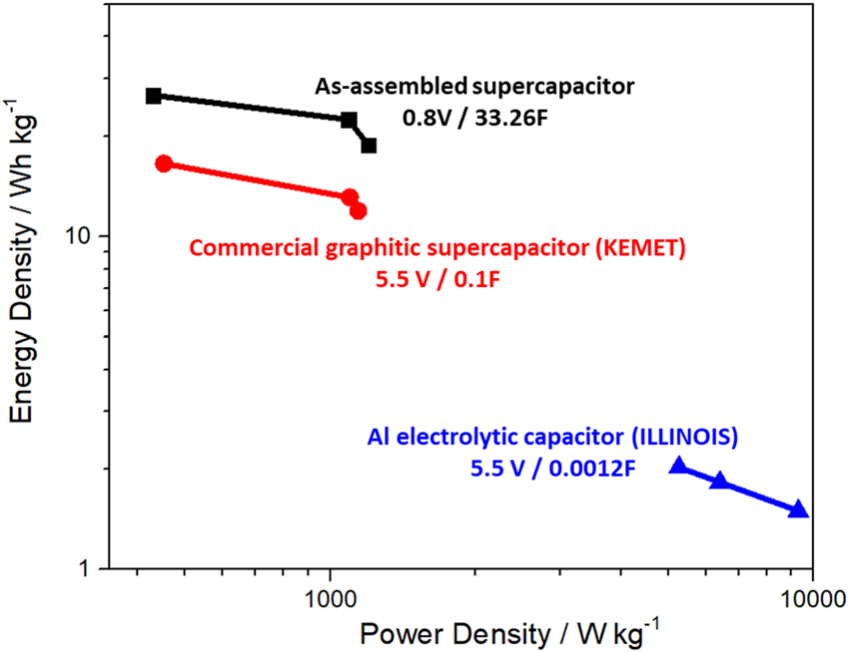
Ragone plots of as-assembled supercapacitor and commercial energy storage devices.

Figure 4 shows a Ragone plot comparing the performance of the as-assembled device to a commercially available Al electrolytic capacitor and KEMET graphitic supercapacitor^30^, as benchmarks to determine the feasibility of the as-assembled supercapacitor (refer Table S1 and Table S2 for the specifications of these devices). The plot shows the gravimetric energy density and power density values for all the devices tested. Although the Al electrolytic capacitor delivered an ultrahigh power density, its energy density was ~50 times lower than that of the stacked device. In comparison, although the EDLC stores energy in much the same way as a traditional capacitor, by means of charge separation, supercapacitors can store substantially more energy (per unit mass or volume) than a conventional capacitor because a large amount of charge can be stored on the highly extended electrode surface area created by the large number of pores within the high-surface-area electrode material^31^. Remarkably, compared to the KEMET graphitic supercapacitor, the as-assembled device exhibited nine times more energy density with a comparable power density. Although both of them employed the electrochemical double layer storage mechanism, the relatively high energy density of the as-assembled supercapacitor may have been contributed by the unique structure of the GNPs, where the interstitial network of agglomerated GNPs provided a less tortuous diffusion path for electrolyte ions to be stored in this reservoir^21,32^. To sum up, the as-assembled device outperformed the selected commercial energy storage devices. The adoption of the extensive casing procedures employed in commercial devices, forming a closed and compact system, could greatly reduce problems such as the evaporation of the aqueous electrolyte over time and the unwanted reaction of the supercapacitor’s components with the atmosphere, enabling the GNP-based supercapacitor to be competitive in the market.

**Figure 5:**
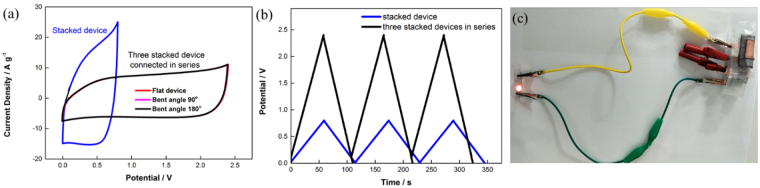
(**a**) CV profiles collected at different bending angles at 100 mV s^−1^. (**b**) GCD curves at 3 A g^−1^ of a stacked supercapacitor and three stacked supercapacitors in series. (**c**) Image of a red LED powered by three 180° bent stacked supercapacitors in series.

To demonstrate the potential usefulness of the as-assembled supercapacitor, three stacked devices were connected in series to make a tandem device. The electrochemical performances of this tandem device were evaluated using CV and GCD measurements (Figure S2). As shown in Fig. 5a,b, the potential window was extended from 0.8 V for the stacked device to 2.4 V for the tandem device. Meanwhile, the produced current (represented by the area under the CV curves) and charge/discharge time at the same current density were essentially unchanged for the tandem device and stacked device, indicating that the capacitive performance of each supercapacitor unit was well retained in the tandem device. Moreover, from the CV profiles recorded in Fig. 5a, it can be seen that different bending angles do not affect the CV curves of the tandem device, demonstrating that it can be bent without affecting the structural integrity of the device. The flexibility of the as-assembled supercapacitor, which enabled it to withstand stress at different curvatures, could be attributed to the exceptional mechanical and electrical robustness of the highly interconnected 2D network structure of GNPs and the favorable interfacial electrochemical behaviors of the GNPs in the Na_2_SO_4_ liquid electrolyte^33^. Lastly, the tandem device, bent at 180°, was used to light up a red light-emitting diode (Fig. 5c) for 40 s after charging for 60 s, which proved the concept of a flexible energy storage device, and most importantly demonstrated the practical potential of the stacked device, where a custom-made supercapacitor with specific voltage and capacitance ratings can be formed using parallel and series configurations.

## Conclusion

This work reported findings from the design, stacking, and testing of supercapacitors. The morphology and electrochemical properties of the GNP electrode material were examined using FESEM, TEM, CV, GCD, and EIS. Through the contributions of the unique properties of the GNP active materials, high ion-diffusion rate of the aqueous Na_2_SO_4_ electrolyte, and sealed and intimate configuration, the equivalent series resistance (ESR) of this stacked device remained low at 0.50 Ω, with good inherent capacitive behaviors. The energy density of the stacked device increased fourfold in magnitude to 24.64 Wh kg^−1^ (vs. 6.28 Wh kg^−1^) without losing its power density of 402 W kg^−1^ (vs. 403 W kg^−1^) compared to a single cell device. It is expected that the design and testing results reported here could form the basis for future commercial applications. Furthermore, the fact that the stacked device outperformed the selected commercially available electronic devices (KEMET and ILLINOIS) and the simplicity of the fabrication process reported here have further reinforced the feasibility of using GNP-based supercapacitors for commercial upscaling, leading to the future production of high-energy-density supercapacitors.

## Experimental

### Materials

Expandable graphite powder was obtained from Ashbury Graphite Mills Inc. (code no. 3772). Polyvinylidene fluoride (PVDF), with an average Mw of 180,000 by GPC, and 99% 1-methyl-2-pyrrolidione (NMP) were purchased from Sigma Aldrich, Malaysia. Sodium sulphate (Na_2_SO_4_) was purchased from Merck, Germany, and 55 mm qualitative filter paper was purchased from Advantec, Toyo Roshi Kaisha, Ltd., Japan. Copper foil with a thickness of 0.025 mm was purchased from Goodfellow Cambridge, England. Double distilled water was used throughout the experiment.

### Material Characterization

The surface morphologies were analyzed using a field emission scanning electron microscope (FEI Nova NanoSEM 400 operated at 20.0 kV) equipped with an energy-dispersive X-ray (EDX) accessory and an HT7701 Hitachi transmission electron microscope (TEM). The crystalline structures of the products were identified by an X-ray diffraction (XRD) analysis using a D8 Advance (Bruker, Karlsruhe, Germany) automated X-ray diffractometer system with Cu−Kα radiation at 40 kV and 40 mA ranging from 10° to 80° at room temperature.

### Preparation of Graphene Nanoplatelets (GNPs)

GNPs were produced by the thermal exfoliation of expandable graphite. The expandable graphite was subjected to a thermal shock at 950 °C for 10 s. Then, the obtained thermally exfoliated graphite was sonicated for 1 h using a probe sonicator to obtain GNPs.

### Assembly of Supercapacitor

**Figure 6:**
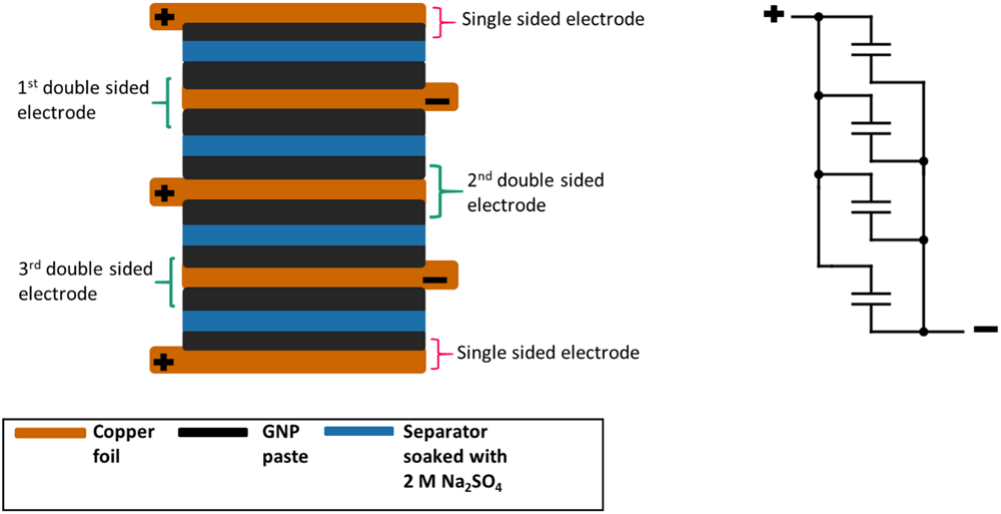
Schematic presentation of multi-celled supercapacitor. On the right is its circuit diagram.

The copper foils were pre-treated by sonication in ethanol, followed by distilled water, and vacuum dried in an oven for 30 min. A slurry paste was formed by mixing GNPs with PVDF in NMP solvent. The paste was doctor bladed on both sides of the clean copper foils, and vacuum dried for 4 h. The mass of each electrode was 0.015 g with a surface area of 2 cm * 2 cm. A supercapacitor was assembled by stacking the copper foils coated with GNPs, which were separated by microporous papers immersed in an aqueous electrolyte consisting of 2 M Na_2_SO_4_. A schematic illustration of the assembly of the supercapacitor is shown in Fig. 6.

### Electrochemical Measurements

Electrochemical measurements for the supercapacitors were carried out using a Princeton potentiostat/galvanostat controlled by Versa Studio software, employing a two-electrode cell system. The electrochemical impedance spectrum (EIS) analyses were carried out in a frequency range of 0.01 Hz to 30 kHz at an open circuit potential with an alternating current perturbation of 5 mV. The capacitance value (C_T_) was calculated from the galvanostatic discharge curve using Eq. 1.

1 $${{\rm{C}}}_{{\rm{T}}}=\frac{{\rm{I}}{\rm{\Delta }}\,{\rm{dt}}}{{\rm{\Delta }}\,{\rm{dV}}}({\rm{F}})$$

where I is the constant discharge current (A g^−1^); t is the discharge time (s); and V is the potential window during the discharge process after the IR drop (V).

The specific energy density (E) and power density (P) are calculated according to Eqs 2 and 3, respectively.

2 $${\rm{E}}=\frac{{{\rm{C}}}_{{\rm{T}}}{\rm{\Delta }}\,{{\rm{V}}}^{2}\,}{2{\rm{m}}}({\rm{Wh}}\,{{\rm{kg}}}^{-1})$$

where C_T_ is the total capacitance calculated from the discharge curve (F); V is the potential window during the discharge process after the IR drop (V); and m is the total mass of the electrode materials (g).

3 $${\rm{P}}=\frac{{\rm{E}}\,}{{\rm{t}}}({\rm{W}}\,{{\rm{kg}}}^{-1})$$

where E is the specific energy density (Wh kg^−1^); and t is the discharge time (s).

The electrochemical measurements of the commercial devices were carried out using the same methods.

### Data availability

Data generated or analysed during this study are included in this published article.

## Electronic supplementary material

Supplementary Information
