# Author Correction: Electrochemical Performance of Supercapacitor with Stacked Copper Foils Coated with Graphene Nanoplatelets

**DOI:** 10.1038/s41598-018-28897-7

**Published:** 2018-07-12

**Authors:** S. L. Chiam, H. N. Lim, S. M. Hafiz, A. Pandikumar, N. M. Huang

**Affiliations:** 1 Department of Chemistry, Faculty of Science, Universiti Putra Malaysia, 43400 UPM Serdang, Selangor Malaysia; 2 Materials Synthesis and Characterization Laboratory, Institute of Advanced Technology, Universiti Putra Malaysia, 43400 UPM Serdang, Selangor Malaysia; 3 Electrochemical Materials Science and Functional Materials Division, CSIR-Central Electrochemical Research Institute, Karaikudi, 630003 India; 4 New Energy Science & Engineering Programme, University of Xiamen Malaysia, Jalan SunSuria, Bandar SunSuria, 43900 Sepang, Selangor Darul Ehsan Malaysia

Correction to: *Scientific Reports* 10.1038/s41598-018-21572-x, published online 15 February 2018

This Article contains a typographical error in the Results and Discussion section where,

“This value is 213 times higher compared to graphitic powder (2.78 m^2^/g) 6, indicating that graphite was significantly exfoliated into few graphitic layers (2630/591 ≈ 4) by thermal exfoliation.”

should read:

“This value is 213 times higher compared to graphitic powder (2.78 m^2^/g)^6^, indicating that graphite was significantly exfoliated into few graphitic layers (2630/591 ≈ 4) by thermal exfoliation.”

In addition, this Article contains an error in the order of the Figures. Figures 2 and 3 were published as Figures 3 and 2 respectively. The correct Figures 2 and 3 appear below as Figures 1 and 2 respectively.

**Figure 1:**
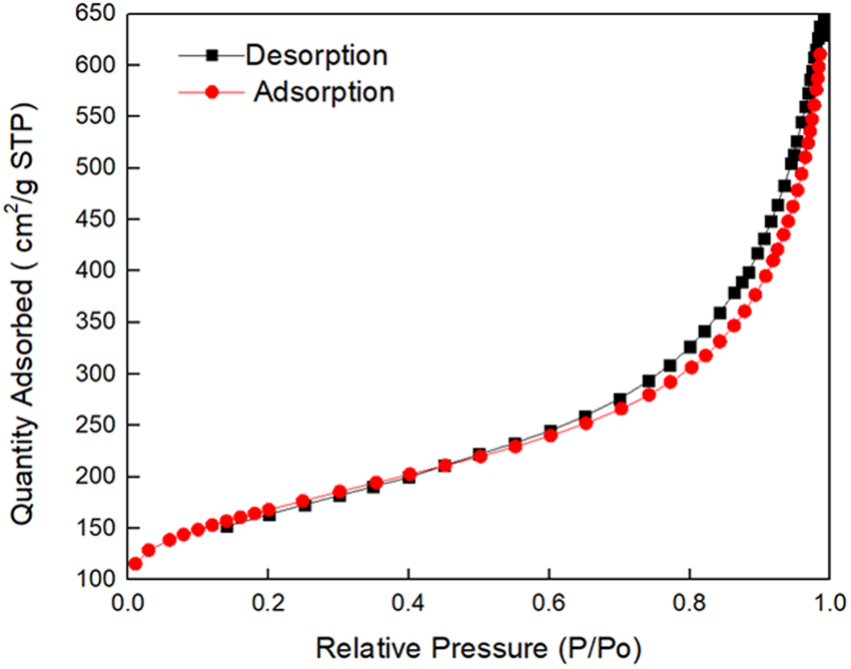
Nitrogen adsorption and desorption isotherms for GNPs.

**Figure 2:**
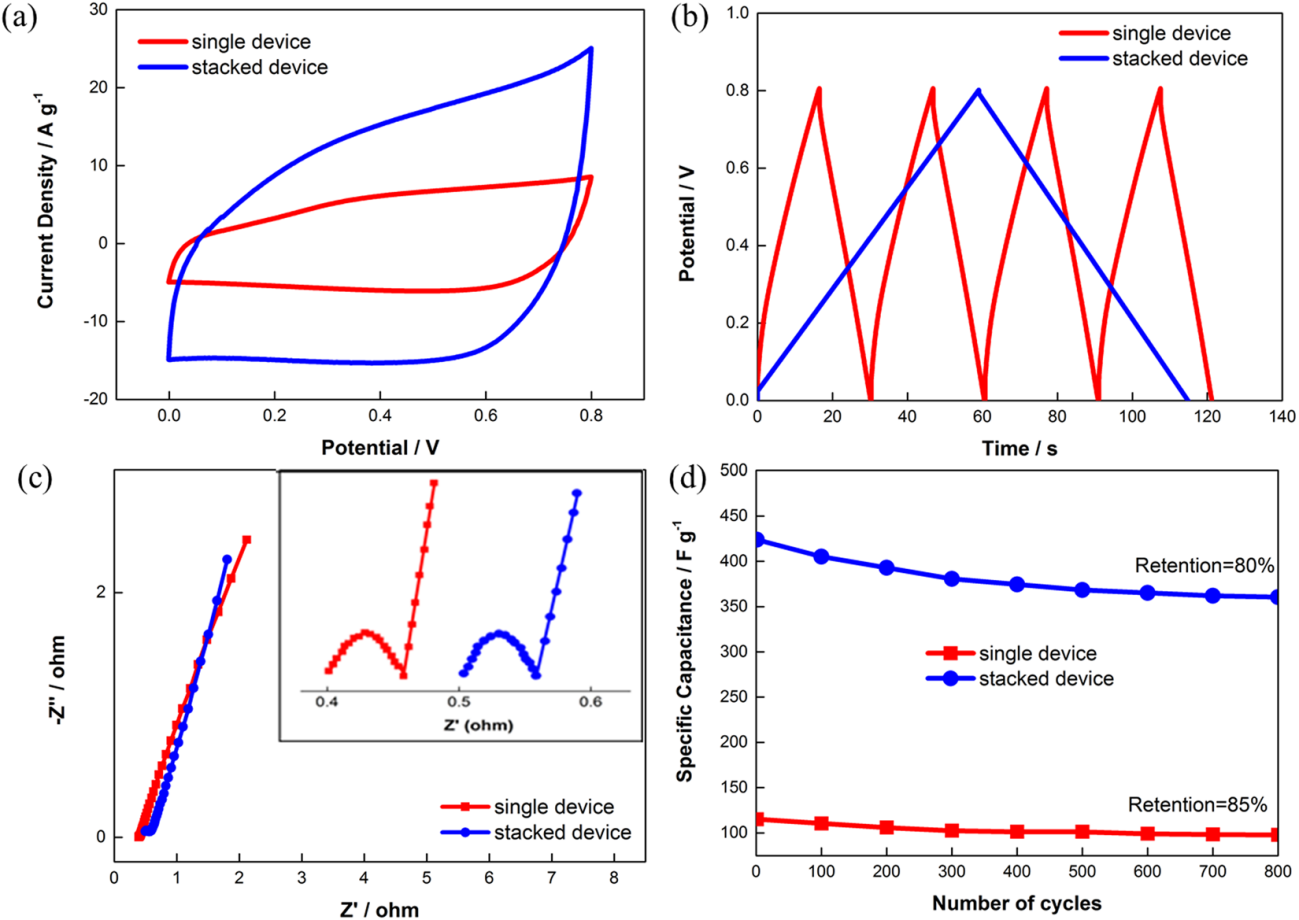
Electrochemical performance of stacked device against single device: (**a**) CV profiles at 100 mV s^−1^, (b) GCD profiles at 3 A g^−1^, (c) Nyquist plot with inset showing magnified version at low-frequency region, and (d) performance durability test at 3 A g^−1^.

