# Supplementary material for: Electrochemical Performance of Supercapacitor with Stacked Copper Foils Coated with Graphene Nanoplatelets
Source: Sci Rep. 2018 Feb 15;8:3093. doi: 10.1038/s41598-018-21572-x (PMC5814563; doi:10.1038/s41598-018-21572-x)
Supplement: Supplementary file 1 — Supplementary Information [file 41598_2018_21572_MOESM1_ESM.docx]

**Electrochemical Performance of Supercapacitor with Stacked Copper Foils Coated with Graphene Nanoplatelets**

S.L. Chiam^1^, H.N. Lim^1,2,^*, S.M. Hafiz^2,^*, A. Pandikumar^3^, N.M. Huang^4,^*

**^1^**Department of Chemistry, Faculty of Science, Universiti Putra Malaysia, 43400 UPM Serdang, Selangor, Malaysia

^2^Materials Synthesis and Characterization Laboratory, Institute of Advanced Technology, Universiti Putra Malaysia, 43400 UPM Serdang, Selangor, Malaysia

^3^Electrochemical Materials Science and Functional Materials Division, CSIR-Central Electrochemical Research Institute, Karaikudi-630003, India

^4^New Energy Science & Engineering Programme, University of Xiamen Malaysia, Jalan SunSuria, Bandar SunSuria, 43900 Sepang, Selangor Darul Ehsan, Malaysia

*Corresponding author: [janetlimhn@gmail.com](mailto:janetlimhn@gmail.com), [syedhafiz27@gmail.com](mailto:syedhafiz27@gmail.com), huangnayming@xmu.edu.my

**
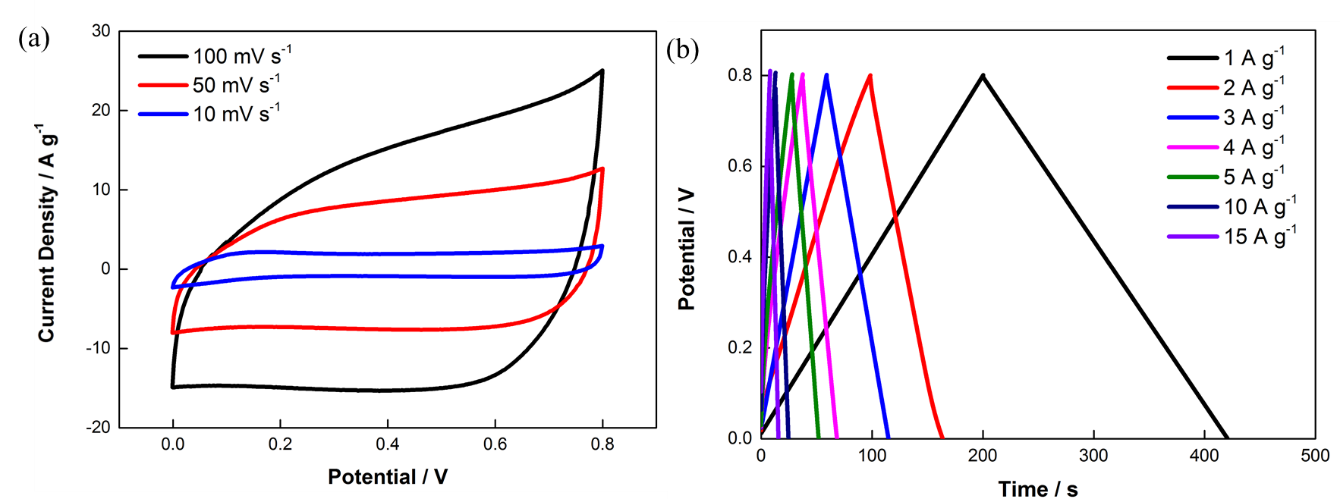
**

**Figure S1.** Electrochemical performances of stacked device. (a) CV curves at scan rates of 10, 50, and 100 mV/s. (b) GCD curves at various current densities.


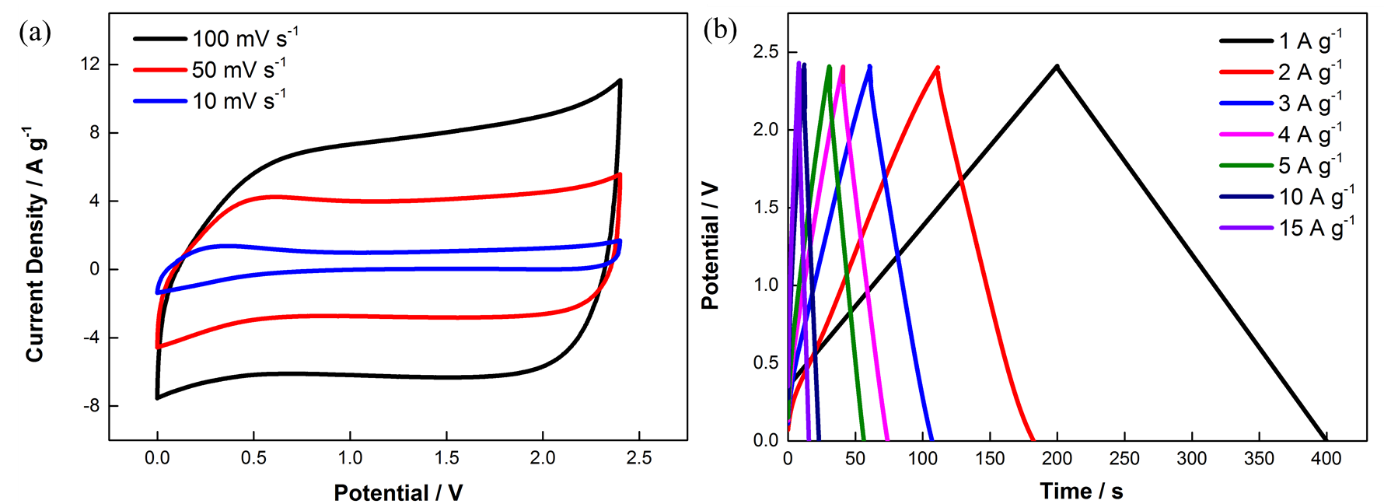


**Figure S2.** Electrochemical performances of three stacked devices in series. (a) CV curves at scan rates of 10, 50, and 100 mV/s. (b) GCD curves at various current densities.

**Table S1.** General information and specifications of Al electrolytic ILLINOIS capacitor (Manufacturer Part Number: 128KXM6R3MLQ).

|  | Specifications |
| --- | --- |
| Description | Radial cylindrical capacitor |
| Capacitance | 0.0012 F |
| Voltage  Temperature Range | 6.3V  -55/+105 °C |

**Table S2.** General information and specifications of KEMET commercial supercapacitor (Manufacturer Part Number: FT0H105ZF).

|  | Specifications |
| --- | --- |
| Description | Radial cylindrical capacitor |
| Capacitance | 0.1 F |
| Voltage | 5.5 V |
| Temperature range | -40/+85 °C |
